# Development and validation of web calculators to predict early recurrence and long-term survival in patients with duodenal papilla carcinoma after pancreaticoduodenectomy

**DOI:** 10.1186/s12885-023-11632-5

**Published:** 2023-11-20

**Authors:** Guangsheng Yu, Shuai Xu, Junjie Kong, Jingyi He, Jun Liu

**Affiliations:** Department of Liver Transplantation and Hepatobiliary Surgery, Shandong Provincial HospitalAffiliated to, Shandong First Medical University , 324 Jingwu Road, Jinan, 250021 Shandong China

**Keywords:** Duodenal papilla carcinoma, Nomogram, Web calculator, Early recurrence, Long-term survival

## Abstract

**Background:**

Duodenal papilla carcinoma (DPC) is prone to relapse even after radical pancreaticoduodenectomy (PD) (including robotic, laparoscopic and open approach). This study aimed to develop web calculators to predict early recurrence (ER) (within two years after surgery) and long-term survival in patients with DPC after PD.

**Methods:**

Patients with DPC after radical PD were included. Univariate and multivariate logistic regression analyses were used to identify independent risk factors. Two web calculators were developed based on independent risk factors in the training cohort and then tested in the validation cohort.

**Results:**

Of the 251 patients who met the inclusion criteria, 180 and 71 patients were enrolled in the training and validation cohorts, respectively. Multivariate logistic regression analysis revealed that tumor size [Odds Ratio (OR) 1.386; 95% confidence interval (CI) 1070–1.797; *P* = 0.014]; number of lymph node metastasis (OR 2.535; 95% CI 1.114–5.769; *P* = 0.027), perineural invasion (OR 3.078; 95% CI 1.147–8.257; *P* = 0.026), and tumor differentiation (OR 3.552; 95% CI 1.132–11.152; *P* = 0.030) were independent risk factors for ER. Nomogram based on the above four factors achieved good C-statistics of 0.759 and 0.729 in predicting ER in the training and the validation cohorts, respectively. Time-dependent ROC analysis (timeROC) and decision curve analysis (DCA) revealed that the nomogram provided superior diagnostic capacity and net benefit compared with single variable.

**Conclusions:**

This study developed and validated two web calculators that can predict ER and long-term survival in patients with DPC with high degree of stability and accuracy.

**Supplementary Information:**

The online version contains supplementary material available at 10.1186/s12885-023-11632-5.

## Background

Duodenal papilla carcinoma (DPC) is a malignant tumor occurring in the duodenal papilla region (including the intrapapillary bile duct and pancreatic duct) and accounts for about 60% of primary duodenal malignant tumors [1, 2]. Compared with pancreatic ductal adenocarcinoma (PDAC) and distal cholangiocarcinoma (DCC), DPC has a higher resection rate and a better long-term prognosis [3]. 

Radical pancreaticoduodenectomy (PD) represents the main surgical strategy for the treatment of DPC, with a 5-year survival rate of about 50% [3]. However, due to the difference in tumor histological grade, tumor size and surrounding tissue invasion, some patients with DPC are still prone to relapse after surgery, which seriously threatens the long-term survival of these patients [3, 4]. Multiple studies have shown that tumor size, lymph node metastasis, tumor differentiation, perineural invasion and TNM stage are independent prognostic factors affecting the long-term survival of patients with DPC [5–7]. However, there is no prediction model that combines these independent prognostic factors to predict early recurrence (ER) and overall survival (OS) of patients with DPC, so as to provide objective evaluation indicators and clinical references [8, 9].

Therefore, in the present study, we retrospectively analyzed clinical and follow-up data from patients with DPC at our center to screen for independent prognostic factors affecting ER and long-term survival. In addition, the web-based calculators for ER and long-term survival in DPC patients were developed following the predictive models to help clinicians screen high-risk patients with poor prognosis and timely adjust corresponding treatment and follow-up strategies to improve the long-term survival outcomes in these patients.

## Methods

### Study population

Clinical pathology results and follow-up data were included for patients with DPC who underwent radical PD at the Department of Liver Transplantation and Hepatobiliary Surgery of Shandong Provincial Hospital between January 2011 and October 2020. This study was approved by the Medical Ethics Committee of Shandong Provincial Hospital (No.2022–178), and all patients gave informed consent and signed written informed consent.

### Inclusion and exclusion criteria

The pre-operative resectability of the DPC was evaluated under the multi-disciplinary team (MDT) strategy, and the diagnosis was confirmed based on the post-operative histopathology results. The inclusion criteria were as follows: (1) age ≥ 18 years; (2) DPC was confirmed by pathological report; (3) radical PD was performed and R0 resection was confirmed by postoperative pathology; (4) the American Society of Anesthesiologists (ASA) staging was I-III; (5) no neoadjuvant therapy (NAT) was received before operation. Exclusion criteria were as follows: (1) death due to complications or other causes other than DPC; (2) incomplete data or lost to follow-up; (3) accompanied by other malignant tumors; (4) the presence of distant metastasis.

### Follow up after resection

Patients were followed up every three to six months after surgery through outpatient reviews or telephone interviews. The results of follow-up were recorded until the patient died or was lost to follow-up. Examination during follow-up included enhanced abdominal and pelvic CT and/or MRI scans, chest X-rays, and tumor marker screening, among others. Tumor recurrence was determined comprehensively based on imaging and serological findings. Postoperative chemotherapy and regular surveillance were recommended. A standard adjuvant chemotherapy regimen has not been established in China. In our center, the main chemotherapy regimens include gemcitabine-based regimens or combinations of gemcitabine and 5-Fu. The primary endpoint of this study was early recurrence (ER), and the secondary endpoint was overall survival (OS). ER was defined as tumor recurrence within 2 years after radical resection (In the result section of this article, we use the minimum *P*-value method to reverse verify this definition [10, 11]). OS was calculated from the date of PD to either the date of death or the date of the last follow-up [12]. The final follow-up date for this study was October 31, 2022.

### Study variables and definition

All patients underwent standard open or minimally invasive approach (robotic or laparoscopic). The specific surgical procedures and protocols have been reported in detail in our previous studies [13, 14]. Surgical modality was not a focus in this study, as a number of domestic and international studies have demonstrated that a minimally invasive or open approach is not a prognostic factor for long-term survival of patients after surgery [15–18].

Conventional demographic indicators included age, sex, body mass index (BMI), ASA health staging, and personal history. Serological variables include: blood routine, biochemical indicators and tumor markers, among others. Serological indicators were based on the results of the first post-admission examination. Histopathologic findings included tumor size (the largest tumor diameter recorded in the pathological reports), tumor differentiation, surrounding invasion, and lymph node metastasis. The Clavien-Dindo grading system assesses postoperative complications, and severe complications were defined as Clavien-Dindo grade III or higher [19]. R0 resection was defined as the absence of tumor cells at the microscopic margin [20].

### Statistical analysis

Continuous variables conforming to normal distribution were expressed as mean ± standard deviation (SD), and differences between groups were compared by student’s t test; while continuous variables that did not conform to normal distribution were expressed as median (interquartile range, [IQR]), and differences between groups were compared by Mann–Whitney U test [21]. Categorical variables were expressed as frequency (%), and differences between groups were compared by Pearson chi-square test, continuity-corrected chi-square test or Fisher’s exact probability test. Referring to previous studies [11, 22], univariate and multivariate logistic regression analyses were used to determine independent prognostic factors for ER in the training cohort. The DPC-ER nomogram was developed according to the proportion of regression coefficients of independent prognostic factors. The calibration curve, receiver operating characteristic (ROC) curve, time-dependent ROC (timeROC) curve, and decision curve analysis (DCA) were used to evaluate the performances of different models across the two cohorts. The optimal cut-off value for the nomogram score was calculated using the maximum Youden index method. The Kaplan–Meier survival curve was used to describe the long-term survival of patients, and the log-rank test was used to compare the differences between groups. All tests were two-tailed, and *P* < 0.05 was defined as statistically significant. The statistical software we used in the present study includes R software (version 3.6.3, R Foundation for Statistical Computing, Vienna, Austria) and SPSS software (version 25.0, IBM, Armonk, New York, USA). The packages used in the R environment mainly include: “survival”,“survminer”,“rmda”,“nomogramFormula”,“timeROC”,“rms”,“pROC”, “DynNom” and “rsconnect”.

## Results

### Clinical characteristics of patients

A total of 251 patients met the inclusion and exclusion criteria to be included in the study. All patients were randomized in a ratio of approximately 7:3, with 180 patients enrolled in the training cohort and 71 in the validation cohort (Supplementary Fig. 1). During the follow-up period, 81 (35.2%) patients experienced ER, including 56 (31.1%) patients in the training cohort and 25 (35.2%) patients in the validation cohort (*P* = 0.531). The median RFS of patients in the training cohort was 36.5 (95% confidence interval [CI]: 27.2–45.8) months, and the median OS was 50.2 (95%CI: 41.1–59.3) months. The median RFS in the validation cohort was 27.6 (95%CI: 22.9–32.3) months, and the median OS was 50.5 (95%CI: 25.0–76.0) months. There were no significant differences between the two cohorts in demographic, serological, tumor histopathology characteristics, or long-term survival outcomes. The detailed characteristics of patients with DPC in the training and validation cohorts were presented in Table 1.

**Table 1:** Clinicopathological characteristics of patients with DPC in the training and validation cohorts (*n* = 251)

Variables	Training cohort	Validation cohort	*P* value
	(***n* = 180**)	(***n* = 71**)	
Age ≤ 60 years	101 (56.1)	34 (47.9)	0.239
Gender, Male	97 (53.9)	42 (59.2)	0.450
BMI, kg/m^2^	23.2 (21.1–25.7)	23.8 (21.5–25.9)	0.433
ASA grade ≤ II	154 (85.6)	56 (78.9)	0.197
Biliary infection	46 (25.6)	16 (22.5)	0.617
Biliary drainage	46 (25.6)	20 (28.2)	0.672
Jaundice history	99 (55.0)	37 (52.1)	0.679
Diabetes history	26 (14.4)	8 (11.3)	0.508
Preoperative WBC, 10^9^/L	6.1 (4.8–7.4)	5.9 (4.7–7.4)	0.539
Preoperative ALB, g/L	37.9 (35.4–40.5)	37.8 (35.1–40.8)	0.817
Preoperative TBIL, μmol/L	23.7 (11.9–97.4)	32.4 (9.7–115.5)	0.729
Preoperative glucose, mmol/L	5.4 (4.8–6.0)	5.1 (4.6–6.2)	0.346
Preoperative CA19-9, U/mL	39.9 (11.0–106.8)	29.6 (13.0–76.7)	0.782
Preoperative CA125, U/mL	11.2 (7.9–17.7)	11.7 (8.8–17.8)	0.360
Preoperative CEA, ng/mL	2.2 (1.5–3.3)	2.1 (1.2–3.5)	0.544
Preoperative FAR	0.107 (0.085–0.130)	0.097 (0.083–0.136)	0.624
Preoperative NLR	2.3 (1.7–3.6)	2.1 (1.5–3.3)	0.142
Preoperative PLR	162.0 (124.0–234.3)	151.2 (122.3–204.3)	0.357
Tumor size, cm	2.0 (1.5–3.0)	2.5 (1.8–3.5)	0.211
Without lymph node metastasis	135 (75.0)	53 (74.6)	0.681
Perineural invasion	24 (13.3)	15 (21.1)	0.125
Vascular invasion	7 (3.9)	1 (1.4)	0.543
TNM stage (I/II/III)	86/49/45 (47.8/27.2/25.0)	45/11/15 (63.4/15.5/21.1)	0.060
Poorly differentiated	47 (26.1)	18 (25.4)	0.893
Clavien-Dindo grade ≥ III	23 (12.8)	13 (18.3)	0.260
Early recurrence	56 (31.1)	25 (35.2)	0.531
Adjuvant chemotherapy	87 (48.3)	38 (53.5)	0.459
Follow-up, months	37.5 (18.8–54.5)	33.8 (14.7–47.8)	0.051
Median RFS (95% CI), months	36.5 (27.2–45.8)	27.6 (22.9–32.3)	0.182
Median OS (95% CI), months	50.2 (41.1–59.3)	50.5 (25.0–76.0)	0.288

Abbreviation: *DPC* Duodenal papilla carcinoma, *IQR* Interquartile range, *BMI* Body mass index, *ASA grade* American Society of Anesthesiologists physical status classification, *WBC* White blood cell, *ALB* Albumin, *TBIL* Total bilirubin, *CA19-9* Carbohydrate antigen19-9, *CEA* Carcinoembryonic antigen, *CA125* Carbohydrate antigen125, *FAR* Fibrinogen-to-albumin ratio, *NLR* Neutrophil–lymphocyte ratio, *PLR* Platelet to lymphocyte ratio, *RFS* Recurrence free survival, *OS* Overall survival, *CI *Confidence interval

Data are presented as n (%) or median (IQR); Bold text hinted that these variables were statistically significant

Table 2 compared the clinical and follow-up data for 81 patients with ER and 170 patients without ER. The results demonstrated that patients with ER had a higher proportion of lymph node metastasis (39.5% vs. 18.2%, *P* = 0.001), peripheral nerve invasion (28.4% vs. 9.4%, *P* < 0.001), TNM stage III (35.8% vs. 18.2%, *P* = 0.005) and poorly differentiated tumors (38.3% vs. 20.0, *P* = 0.001). Additionally, tumors were relatively larger in the ER group (2.5 cm vs. 2.0 cm, *P* = 0.003). The median RFS of patients in the ER group was 11.4 (95%CI: 10.1–12.7) months, and the median OS was 21.9 (95%CI: 18.2–25.6) months, both significantly shorter than those in the non-ER group (both *P* values < 0.001). Additional demographic, serological and histopathological results for both groups are detailed in Table 2.

**Table 2:** Clinicopathological characteristics and surgical outcomes between patients in DPC-ER group and Non-DPC-ER group

Variables	Non-DPC-ER group	DPC-ER group	*P* value
	(***n*** = 170)	(*n*** = 81**)	
Age ≤ 60 years	92 (54.1)	43 (53.1)	0.878
Gender, Male	97 (57.1)	42 (51.9)	0.438
BMI, kg/m^2^	23.8 (21.4–26.1)	22.7 (20.8–24.6)	**0.023**
ASA grade ≤ II	144 (84.7)	66 (81.5)	0.518
Biliary infection	41 (24.1)	21 (25.9)	0.756
Biliary drainage	43 (25.3)	23 (28.4)	0.602
Jaundice history	90 (52.9)	46 (56.8)	0.567
Diabetes history	23 (13.5)	11 (13.6)	0.991
Preoperative WBC, 10^9/L	6.2 (4.8–7.4)	5.9 (4.7–7.1)	0.519
Preoperative ALB, g/L	37.9 (35.6–40.4)	37.5 (34.5–40.8)	0.728
Preoperative TBIL, μmol/L	24.0 (11.9–99.8)	31.5 (9.7–104.8)	0.752
Preoperative glucose, mmol/L	5.3 (4.7–6.0)	5.2 (4.8–6.2)	0.733
Preoperative CA19-9, U/mL	31.8 (10.8–82.1)	46.9 (15.2–133.2)	**0.048**
Preoperative CA125, U/mL	11.0 (8.1–17.7)	12.1 (9.0–18.2)	0.267
Preoperative CEA, ng/mL	2.2 (1.4–3.4)	2.0 (1.3–3.1)	0.535
Preoperative FAR	0.106 (0.084–0.127)	0.108 (0.086–0.139)	0.363
Preoperative NLR	2.3 (1.6–3.6)	2.4 (1.6–3.4)	0.983
Preoperative PLR	154.8 (116.4–218.3)	162.3 (131.6–230.0)	0.258
Tumor size, cm	2.0 (1.5–3.0)	2.5 (1.8–4.0)	**0.003**
Lymph node metastasis	31 (18.2)	32 (39.5)	**0.001**
Perineural invasion	16 (9.4)	23 (28.4)	** < 0.001**
Vascular invasion	5 (2.9)	3 (3.7)	0.748
TNM stage (I/II/III)	99/40/31 (58.2/23.5/18.2)	32/20/29 (39.5/24.7/35.8)	**0.005**
Poorly differentiated	34 (20.0)	31 (38.3)	**0.001**
Clavien-Dindo grade ≥ III	26 (15.3)	10 (12.3)	0.533
Adjuvant chemotherapy	85 (50.0)	40 (49.4)	0.927
Median RFS (95% CI), months	62.5 (NA-NA)	11.4 (10.1–12.7)	** < 0.001**
Median OS (95% CI), months	NA (NA-NA)	21.9 (18.2–25.6)	** < 0.001**

Abbreviation: *DPC *Duodenal papilla carcinoma, *ER* Early recurrence, *IQR* Interquartile range, *BMI* Body mass index, *ASA grade* American Society of Anesthesiologists physical status classification, *WBC* White blood cell, *ALB* Albumin, *TBIL* Total bilirubin, *CA19-9* Carbohydrate antigen19-9, *CEA* Carcinoembryonic antigen, *CA125* Carbohydrate antigen125, *FAR* Fibrinogen-to-albumin ratio, *NLR* Neutrophil–lymphocyte ratio, *PLR* Platelet to lymphocyte ratio, *RFS* Recurrence free survival, *OS* Overall survival, *CI* Confidence interval, *NA* Not available

Data are presented as n (%) or median (IQR); Bold text hinted that these variables were statistically significant

### Validation of ER definitions and ER sites.

As mentioned above, we used the minimum *P*-value method to conduct a minimum *P*-value analysis of postoperative OS in the potential ER group and the non-ER group following previous studies [10, 11]. The results indicated that the *P*-value of log-rank test was the smallest when the cut-off value was 24 months (*P* = 3.596 × 10^–44^) (Supplementary Table 1). Among the 81 ER patients, local recurrence and liver metastasis were the most common, accounting for 19.8% and 48.1%, respectively. See Supplementary Table 2 for details of the ER sites.

### Identification of independent prognostic factors for ER and the development of a prognostic nomogram in the training cohort

**Fig. 1:**
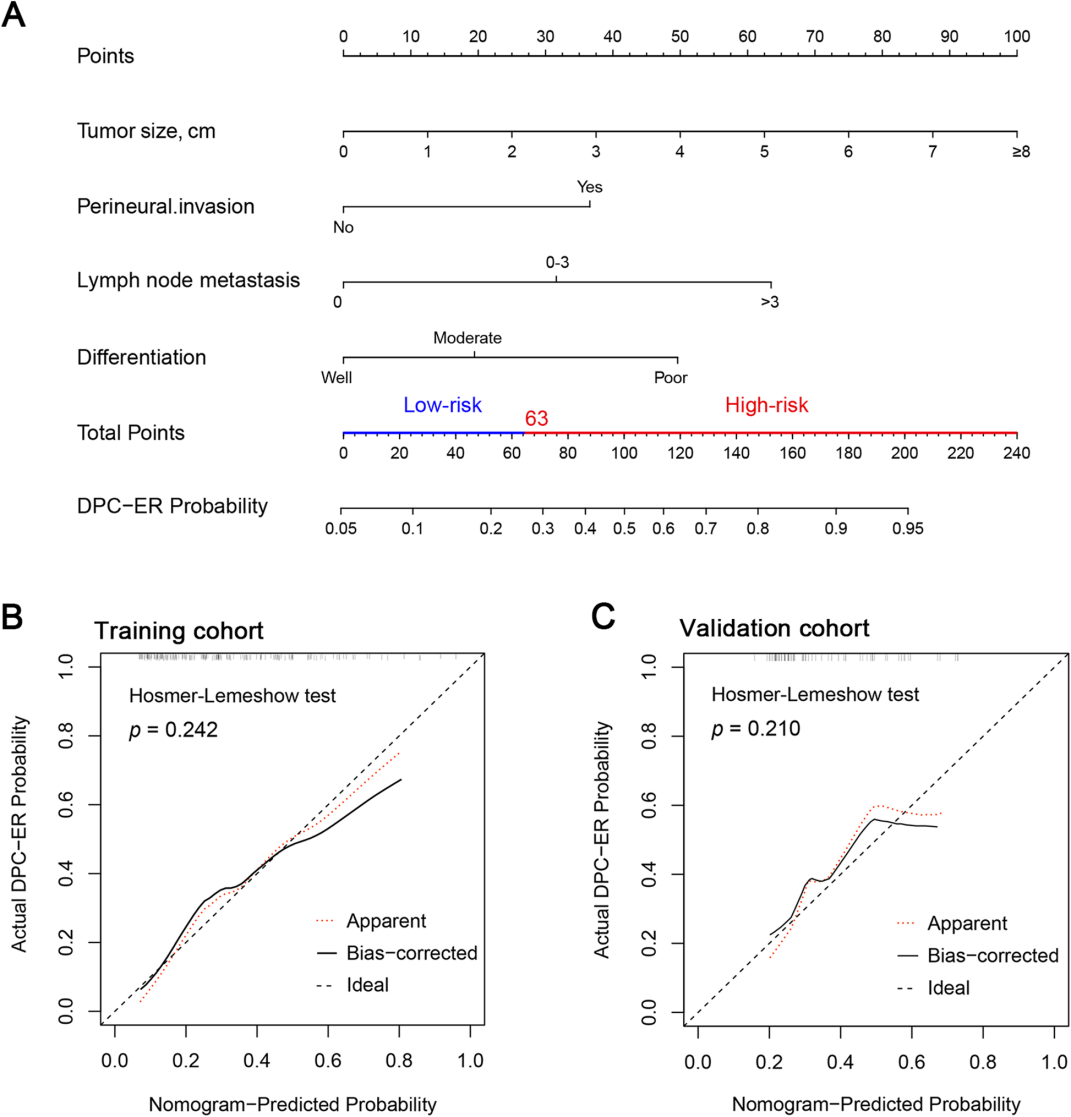
DPC-ER nomogram for predicting early recurrence (ER) of patients with duodenal papilla carcinoma (DPC) after radical pancreaticoduodenectomy (PD) and calibration curves in the two cohorts. [A, DPC-ER nomogram, the optimal cutoff value of nomogram score was 63, low-risk group: nomogram score ≤ 63; high-risk group: nomogram score > 63; B-C, calibration curves in the training and the validation cohorts, respectively.]

Multivariate logistic regression analysis revealed that tumor size (odds ratio [OR]: 1.386; 95% CI: 1.070–1.797; *P* = 0.014), number of lymph node metastasis (OR: 2.535; 95% CI: 1.114–5.769; *P* = 0.027), perineural invasion (OR: 3.078; 95% CI: 1.147–8.257; *P* = 0.026), poorly tumor differentiation (OR: 3.552; 95% CI: 1.132–11.152; *P* = 0.030) were the independent prognostic factors for ER of DPC after radical resection (Table 3). Then, we constructed the prognostic nomogram for predicting ER of DPC based on the above four independent prognostic factors (Fig. 1A).

**Table 3:** Univariable and multivariable logistic analysis for ER in the training cohort

Characteristics	Univariable analysis			Multivariable analysis		
	**B**	**OR (95% CI)**	***P* value**	**B**	**OR (95% CI)**	***P* value**
Age, > 60 vs ≤ 60, years	0.044	1.045 (0.554–1.973)	0.891			
Gender, male vs female	-0.435	0.647 (0.343–1.220)	0.178			
BMI, per kg/m^2^	-0.058	0.943 (0.862–1.032)	0.205			
ASA grade, ≥ III vs ≤ II	0.187	1.205 (0.501–2.899)	0.677			
Biliary infection, yes vs no	0.093	1.098 (0.535–2.250)	0.799			
Biliary drainage, yes vs no	-0.182	0.834 (0.399–1.742)	0.629			
Jaundice, yes vs no	0.126	1.134 (0.600–2.143)	0.698			
Diabetes history, yes vs no	-0.236	0.789 (0.311–2.002)	0.619			
Preoperative WBC, per 10^9/L	0.047	1.048 (0.900–1.220)	0.546			
Preoperative ALB, per g/L	-0.025	0.975 (0.898–1.059)	0.552			
Preoperative TBIL, per μmol/L	0.000	1.000 (0.996–1.004)	0.983			
Preoperative glucose, per mmol/L	-0.056	0.945 (0.769–1.162)	0.593			
Preoperative CA19-9, per U/mL	0.002	1.002 (1.000–1.003)	**0.013**	0.001	1.001 (0.999–1.002)	0.229
Preoperative CA125, per U/mL	0.009	1.009 (0.983–1.037)	0.495			
Preoperative CEA, per ng/mL	-0.002	0.998 (0.981–1.015)	0.792			
Preoperative FAR*100	0.054	1.056 (0.973–1.146)	0.195			
Preoperative NLR	0.003	1.003 (0.943–1.067)	0.927			
Preoperative PLR	0.001	1.001 (0.999–1.003)	0.393			
Tumor size, per cm	0.326	1.385 (1.097–1.749)	**0.006**	0.327	1.386 (1.070–1.797)	**0.014**
Number of lymph node metastasis						
0–3 vs 0	1.077	2.936 (1.400–6.157)	**0.004**	0.930	2.535 (1.114–5.769)	**0.027**
> 3 vs 0	1.822	6.182 (1.083–35.297)	**0.040**	1.754	5.780 (0.848–39.389)	0.073
Perineural invasion, yes vs no	1.335	3.800 (1.568–9.210)	**0.003**	1.124	3.078 (1.147–8.257)	**0.026**
Vascular invasion, yes vs no	0.530	1.698 (0.367–7.854)	0.498			
Differentiation						
Moderate vs Well	0.972	2.643 (0.999–6.995)	0.050	0.492	1.635 (0.572–4.677)	0.359
Poor vs Well	1.777	5.910 (2.099–16.636)	**0.001**	1.268	3.552 (1.132–11.152)	**0.030**
Clavien-Dindo grade, ≥ III vs ≤ II	-0.281	0.755 (0.281–2.031)	0.578			
Adjuvant chemotherapy, yes vs no	-0.111	0.895 (0.476–1.684)	0.731			

Abbreviation: *DPC* Duodenal papilla carcinoma, *ER* Early recurrence,  *OR* Odds ratio, *B* Coefficient, *CI* Confidence interval, *BMI* Body mass index, *ASA grade* American Society of Anesthesiologists physical status classification, *WBC* White blood cell, *ALB* Albumin, *TBIL* Total bilirubin, *CA19-9* Carbohydrate antigen19-9, *CEA* Carcinoembryonic antigen, *CA125* Carbohydrate antigen125, *FAR* Fibrinogen-to-albumin ratio, *NLR* Neutrophil–lymphocyte ratio, *PLR* Platelet to lymphocyte ratio

Bold text hinted that these variables were statistically significant in univariable or multivariable analyses

### Performance of the DPC-ER nomogram for predicting ER compared to a single variable and TNM stage in training and validation cohorts

**Fig. 2:**
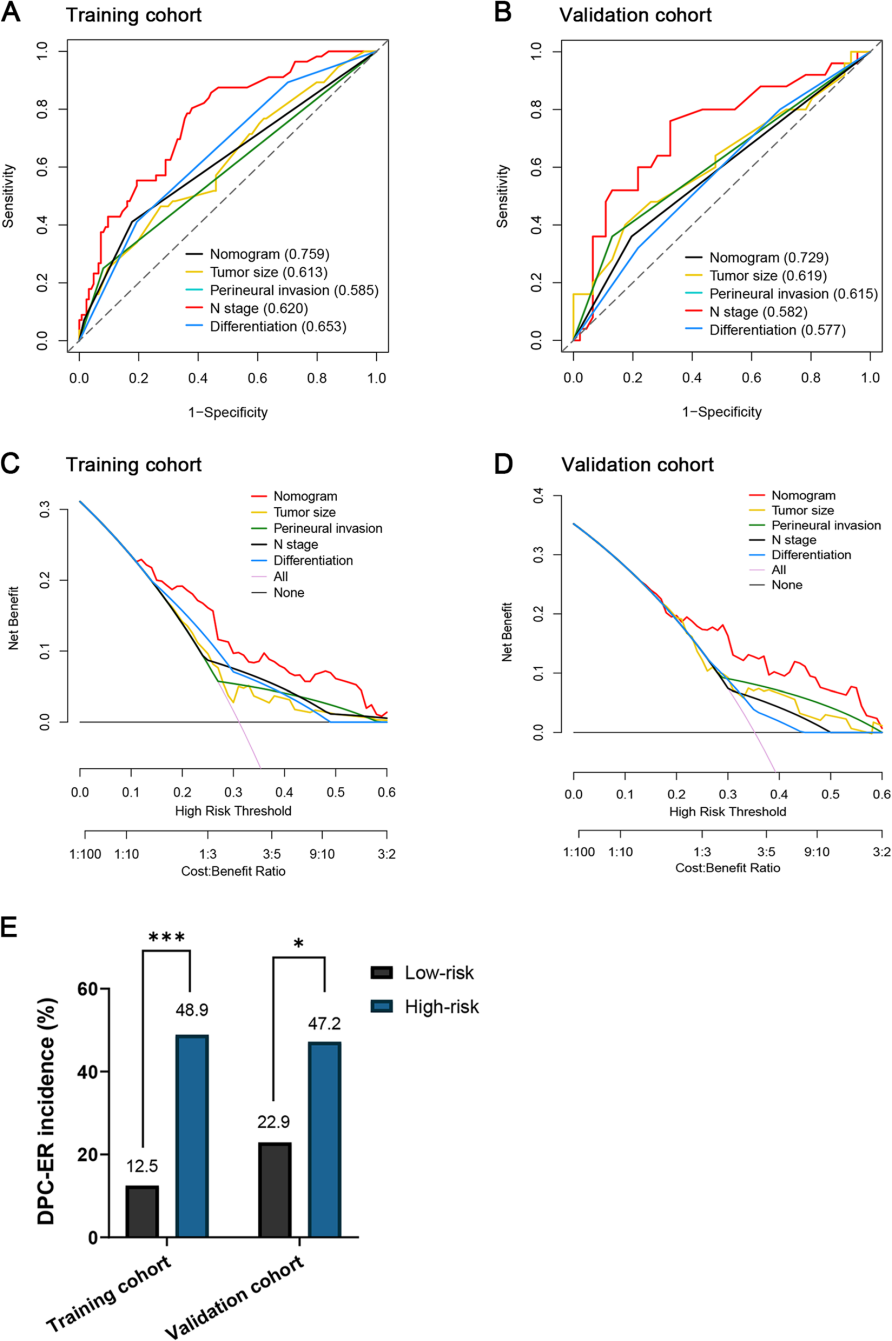
The performance of the DPC-ER nomogram for predicting early recurrence (ER) compared with single variable in the training and validation cohorts. [A-B, receiver operating characteristic (ROC) curve analyses in the training and the validation cohorts, respectively; C-D, decision curve analysis (DCA) in the training and the validation cohorts, respectively; E, comparison of ER rates between patients in low- and high- risk groups]

As shown in Fig. 1B-1C, calibration curves showed that the DPC-ER nomogram fitted well both in the training cohort and the validation cohort (the *P* values of Hosmer–Lemeshow test were 0.242 and 0.210 in the training cohort and the validation cohort, respectively). The C-statistics or the area under the ROC (AUC) curve of the DPC-ER nomogram for ER prediction was 0.759 (95% CI, 0.685–0.832) in the training cohort and 0.729 (95% CI, 0.601–0.856) in the validation cohort, which were both significantly superior to a single variable and TNM stage (all *P* < 0.05, Fig. 2A-2B, Supplementary Fig. 2A-2B, Supplementary Table 3).

Decision curve analysis (DCA) converts complex mathematical models into simple and easy-to-understand graphics for display, in order to intuitively judge the practicability and net benefits of different models [23]. DCA demonstrated that the DPC-ER nomogram provided superior net benefits when compared with a single variable and TNM stage (Fig. 2C-2D and Supplementary Fig. 2C-2D, in the training and validation cohorts, respectively).

The discriminatory ability of the nomogram was further evaluated by dividing patients into two risk groups according to different nomogram scores (the low-risk group with a nomogram score ≤ 63; and the high-risk group with a nomogram score > 63) (Fig. 1A). The results revealed that the high-risk group had a higher ER incidence than the low-risk group in the two cohorts: high-risk group vs. low-risk group, 48.9% vs*.* 12.5% in the training cohort, *P* < 0.001; 47.2% vs. 22.9% in the validation cohort, *P* = 0.032 (Fig. 2E, Supplementary Table 4).

### The long-term survival outcomes of patients in different risk groups

**Fig. 3:**
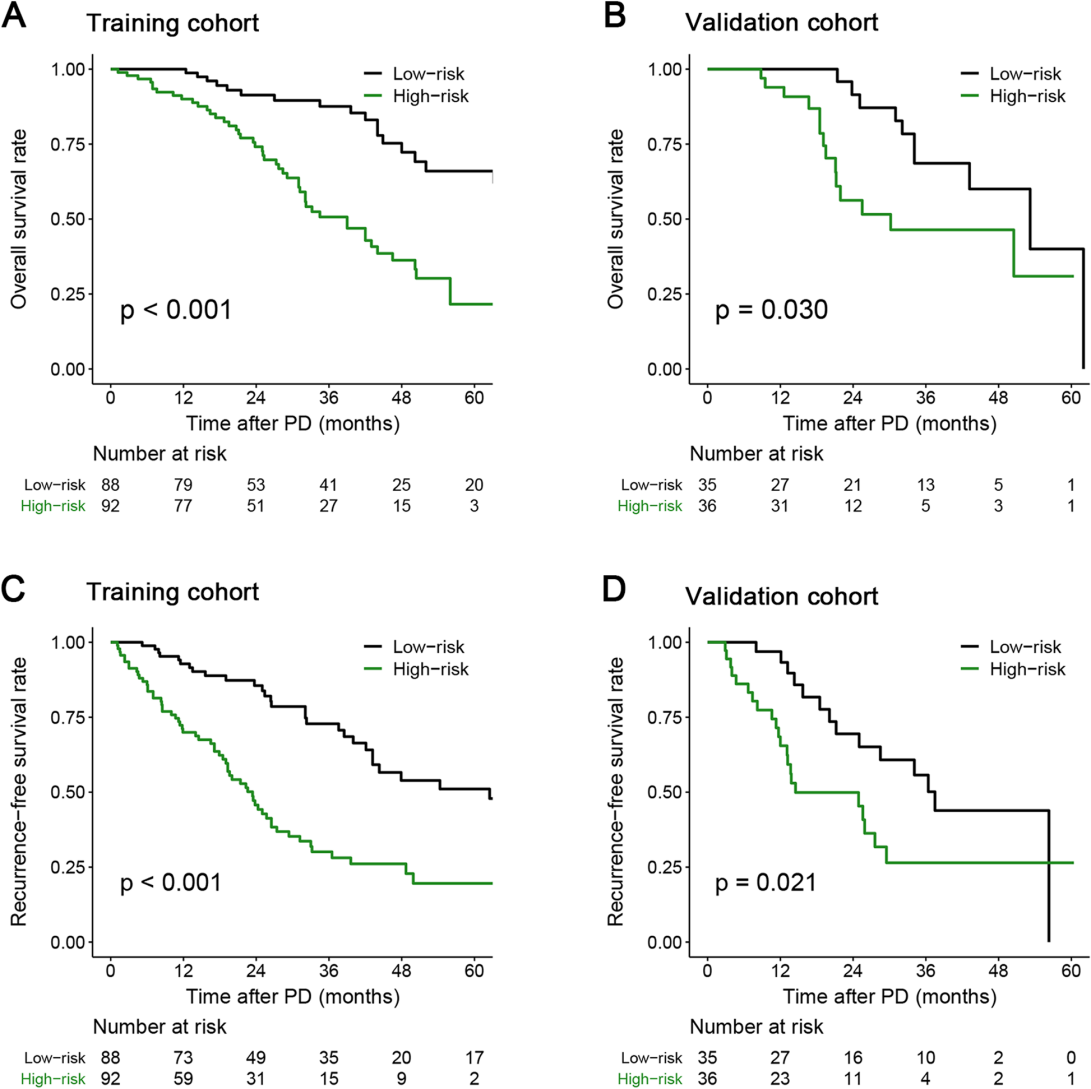
Survival analysis between patients with nomogram score ≤ 63 (low-risk group) and > 63 (high-risk group) in the two cohorts. [A-B, overall survival (OS) between patients in the low-, and high- risk groups in the two cohorts; C-D, recurrence-free survival (RFS) between patients in the low-, and high- risk groups in the two cohorts]

As shown in Fig. 3A-3D, there were significant differences in long-term survival outcomes between different risk groups. The 1-, 3-, 5-year OS rates of patients in the low-risk group were 100.0%, 87.6%, 66.0% in the training cohorts, and 100.0%, 68.6%, 40.0% in the validation cohort, which were all significantly higher than those in the high-risk group: 90.0%, 50.7%, 21.6% in the training cohort, and 93.9%, 46.4%, 30.9% in the validation cohort (*P* < 0.001) (Fig. 3A-3B, Supplementary Table 5). Similar trends in RFS rates were observed in both groups. The detailed results can be seen in Fig. 3C-3D and Supplementary Table 6.

### Performance of the DPC-OS nomogram for predicting OS compared to a single variable in training and validation cohorts

Subsequently, we developed the DPC-OS nomogram for predicting long-term survival outcomes of DPC patients based on the above four independent prognostic factors (Supplementary Fig. 3A). Time-dependent ROC curve (timeROC) analysis was used to further demonstrate the performance of DPC-OS nomogram and single variable in predicting long-term survival outcomes. The area under the timeROC (timeAUCs) of the DPC-OS nomogram for predicting OS within 5 years in the training and validation cohorts were 0.666–0.848 and 0.638–0.953, respectively, which were significantly superior to those of the other four variables (all *P* < 0.001). More details of the timeAUCs of the DPC-OS nomogram and other variables within 5 years are shown in Supplementary Table 7 and Supplementary Fig. 3B-3C.

### Development of the web-based online calculators in predicting ER and OS

To facilitate clinical application, we further converted the DPC-ER nomogram and DPC-OS nomogram into web calculators (Supplementary Fig. 4- Supplementary Fiure 5). The web calculators can be accessed at http://114.115.144.103/dpc and https://abc123-456.shinyapps.io/AAC-OS/ to predict the ER and OS in patients with DPC after PD. The prediction probability can be easily determined by inputting clinical features and reading the output results generated by the webserver.

## Discussion

The duodenal papilla is located at the opening where the pancreatic-bile ducts merge into the duodenum [1, 2]. Tumors growing in this region can present with progressive and painless jaundice at an early stage, offering the possibility of early diagnosis [3, 24]. Compared to distal cholangiocarcinoma and pancreatic head adenocarcinoma, DPC has a higher resection rate and a better long-term prognosis, with a five-year survival rate of up to 50% [3]. PD is by far the preferred strategy for radical resection of DPC, however, even after radical resection, some patients are prone to relapse. In this study, we integrated four independent prognostic factors for DPC to develop and validate a series of nomograms to predict ER and long-term survival for DPC after PD. Both the DPC-ER and DPC-OS nomogram showed higher predictive accuracy and net benefit than single variable (including tumor size, perineural invasion, lymph node metastasis, and tumor differentiation status).

Early tumor recurrence is a common and fatal condition in various malignancies, including DPC, and often indicates a poor prognosis [2, 25, 26]. Although DPC has a better long-term prognosis than pancreatic head adenocarcinoma and distal cholangiocarcinoma, 30% of patients relapse within 2 years of surgery, particularly liver metastasis and local recurrence [3]. During follow-up, therefore, care should be taken to monitor the primary tumor site and liver, and screening for independent prognostic factors associated with tumor recurrence may help guide further treatment of these patients and improve their overall prognosis [3, 12]. Previous studies have shown that tumor size, lymph node metastasis and tumor differentiation status are important prognostic factors affecting the recurrence and long-term survival of DPC after resection [3]. Consistent with previous studies, in the present study, multivariate logistic regression analysis revealed that tumor size, peripheral nerve invasion, lymph node metastasis, and tumor differentiation status were also independent prognostic factors for ER of DPC.

Yoen et al. [27] analyzed preoperative imaging findings of ampullary or papilla carcinoma and found that tumor size was an independent prognostic factor affecting OS and disease-free survival (DFS). Park et al. [28] also indicated that tumor recurrence was significantly affected by tumor diameter. Larger tumor diameters often indicate that the tumor was detected later and has a broader invasion area [27, 28]. Several studies have shown that lymph node metastasis and perineural invasion are the major factors affecting the recurrence and long-term survival of DPC after surgery. de Castro et al. [29] demonstrated that the long-term prognosis of patients was negatively correlated with the number of lymph node metastases. Sakata et al. [30] also found that the number, not the location, of positive regional lymph nodes independently affects long-term survival after resection in patients with ampullary carcinoma. These findings suggest that standardized lymph node dissection may be important in improving long-term outcomes in these patients. Although the incidence of perineural invasion in DPC is lower, perineural invasion often indicates tumor progression and a poorer prognosis [31]. Junrungsee et al. [24] retrospectively analyzed the clinicopathological data of 72 patients with carcinoma of the ampulla of vater (CAV) treated by PD and found that tumor differentiation status was also an important prognostic factor affecting the long-term survival of these patients. Tumor differentiation status is also an important prognostic factor for ER and long-term survival in patients with DPC. Poor tumor differentiation often indicates strong invasion ability and early metastasis [24, 29, 31]. It is worth noting that adjuvant chemotherapy was not an independent prognostic factor for ER and only about half of the patients received adjuvant chemotherapy, given that the cause could be related to psychosocial factors and chemotherapy side effects. The definitive benefit of adjuvant therapy and the optimal choice of the therapeutic schema for DPC patients is unknown [32]. In China, some patients prefer to opt for Chinese herbal remedies.

In this study, we demonstrated the value of these four factors in predicting long-term survival outcomes with DPC and, for the first time, combined these four independent prognostic factors to develop DPC-ER and DPC-OS models for ER and OS prediction. The results indicated that the prediction models exhibited satisfactory prediction performance in both the training and validation cohorts. The DPC nomogram models were more accurate in predicting ER and long-term survival compared to a single variable and TNM stage. In addition, patients were further divided into different risk subgroups according to the DPC-ER nomogram score (high-risk group: nomogram score > 63; low-risk group: nomogram score ≤ 63). The results showed that patients in the high-risk group had significantly higher ER rates than those in the low-risk group, and that patients in the low-risk group had significantly better RFS and OS than those in the high-risk group. Thus, with the DPC predictive models, we can screen high-risk patients for tumor recurrence at an early stage and recommend them for closer post-operative follow-up and timely adjuvant therapy to prolong survival. In addition, we further transform the series nomogram into an online calculator so that it can be used in real-time to provide predictive capabilities in computers or mobile terminals, thus generating a high level of clinical utility [33].

Although the DPC prediction models constructed in this study can effectively screen ER patients and accurately predict their long-term survival after radical resection, several limitations need to be clarified. First, this study is a retrospective study conducted from a single center, and inherent biases are inevitable. In the future, multicenter and prospective studies are needed to validate the predictive power of the web-based calculators. Second, the sample size of this study is relatively small and we will further collect relevant patients in the future to provide more convincing validation results. Third, pathology classification of DPC was not performed in this study due to constraints from existing pathology diagnosis results. Finally, given the fact that the specific adjuvant therapy and outcomes were unknown, further exploration of the clinical efficacy of adjuvant therapy and different adjuvant therapies in patients in high-risk and low-risk subgroups is warranted in the future.

## Conclusions

In conclusion, this study integrated four independent prognostic factors, including tumor size, lymph node metastasis, tumor differentiation status, and perineural invasion, to develop the DPC web calculators for predicting ER and long-term survival of DPC after radical resection. With its high predictive accuracy and net benefit, the web-based calculators can screen high-risk patients prone to relapses, which is expected to help clinicians make individualized clinical decisions and improve overall survival outcomes for these patients.

### Supplementary Information

**Additional file 1: Supplementary figure 1. **Flowchart of our study.** Supplementary table 1. **Evaluated cut-off thresholds for defining DPC-ER based on the overall survival in all DPC patients (*n*=251). **Supplementary table 2. **Recurrence sites in DPC patients with ER after radical pancreaticoduodenectomy. (*n*=81).** Supplementary table 3. **Comparison of different models in predicting DPC-ER.** Supplementary figure 2. **The performance of the DPC-ER nomogram for predicting early recurrence (ER) compared with TNM stage in the training and validation cohorts. **Supplementary table 4. **The DPC-ER incidence between patients with nomogram score ≤ 63 (low-risk group) and >63 (high-risk group) in the training and the validation cohorts.** Supplementary table 5. **Overall survival probability and median survival time between patients with nomogram score ≤ 63 (low-risk group) and >63 (high-risk group) in training and validation cohorts. **Supplementary table 6. **Recurrence-free survival probability and median survival time between patients with nomogram score ≤ 63 (low-risk group) and >63 (high-risk group) in training and validation cohorts. **Supplementary table 7. **Comparison of different models in predicting OS of patients with DPC after PD in training and validation cohorts.** Supplementary figure 4. **The screenshot of DPC-ER web calculator. **Supplementary figure 5. **The screenshot of DPC-OS web calculator. 

## Data Availability

The datasets used and/or analyzed during the current study are available from the corresponding author on reasonable request.

## Abbreviations

DPC: Duodenal papilla carcinoma
PDAC: Pancreatic ductal adenocarcinoma
DCC: Distal cholangiocarcinoma
PD: Pancreaticoduodenectomy
MDT: Multi-disciplinary team
BMI: Body mass index
ASA: American Society of Anesthesiologists
ER: Early recurrence
OS: Overall survival
RFS: Recurrence-free survival
SD: Standard deviation
IQR: Interquartile range
OR: Odds ratio
CI: Confidence interval
ROC: Receiver operating characteristic curve
AUC: Area under the ROC curve
DCA: Decision curve analysis
timeROC: Time-dependent ROC analysis
CAV: Carcinoma of the ampulla of vater

## References

[1] Kim MH, Lee SK, Seo DW, Won SY, Lee SS, Min YI (2001). Tumors of the major duodenal papilla. Gastrointest Endosc..

[2] Lu Y, Li W, Liu G, Yang Y, Xiao E, Mu S, Guo Y, Li D, Yan G (2021). Identification of critical pathways and potential therapeutic targets in poorly differentiated duodenal papilla adenocarcinoma. Cancer Cell Int.

[3] Lian P-L, Chang Y, Xu X-C, Zhao Z, Wang X-Q, Xu K-S (2017). Pancreaticoduodenectomy for duodenal papilla carcinoma: A single-centre 9-year retrospective study of 112 patients with long-term follow-up. World J Gastroentero.

[4] Zhao R, Chang Y, Wang X, Zhang P, Zhang C, Lian P (2018). Pylorus-preserving pancreaticoduodenectomy versus standard pancreaticoduodenectomy in the treatment of duodenal papilla carcinoma. Oncol Lett.

[5] Li H-B, Zhao F-Q, Zhou J (2019). Prognostic Nomogram for Disease-Specific Survival in Patients with Non-metastatic Ampullary Carcinoma After Surgery. Ann Surg Oncol.

[6] Moekotte AL, Malleo G, van Roessel S, Bonds M, Halimi A, Zarantonello L, Napoli N, Dreyer SB, Wellner UF, Bolm L (2020). Gemcitabine-based adjuvant chemotherapy in subtypes of ampullary adenocarcinoma: international propensity score-matched cohort study. Br J Surg.

[7] Moekotte AL, Lof S, Van Roessel S, Fontana M, Dreyer S, Shablak A, Casciani F, Mavroeidis VK, Robinson S, Khalil K (2020). Histopathologic Predictors of Survival and Recurrence in Resected Ampullary Adenocarcinoma: International Multicenter Cohort Study. Ann Surg.

[8] Moekotte A, van Roessel S, Malleo G, Rajak R, Ecker B, Fontana M, Han H, Rabie M, Roberts K, Khalil K (2020). Development and external validation of a prediction model for survival in patients with resected ampullary adenocarcinoma. Eur J Surg Oncol.

[9] Huang X, Huang C, Chen W, Cai J, Gan T, Zhao Y, Liu Q, Liang L, Yin X (2020). Development and validation of a nomogram for predicting overall survival of node-negative ampullary carcinoma. J Surg Oncol.

[10] Sahara K, Tsilimigras D, Kikuchi Y, Ethun C, Maithel S, Abbott D, Poultsides G, Hatzaras I, Fields R, Weiss M (2021). Defining and Predicting Early Recurrence after Resection for Gallbladder Cancer. Ann Surg Oncol.

[11] Groot VP, Gemenetzis G, Blair AB, Rivero-Soto RJ, Yu J, Javed AA, Burkhart RA, Rinkes IHMB, Molenaar IQ, Cameron JL (2019). Defining and Predicting Early Recurrence in 957 Patients With Resected Pancreatic Ductal Adenocarcinoma. Ann Surg.

[12] Xu S, Zhang XP, Zhao GD, Zou WB, Zhao ZM, Hu MG, Gao YX, Tan XL, Liu Q, Liu R. A novel online calculator to predict recurrence risk in patients with distal cholangiocarcinoma after radical pancreaticoduodenectomy. J Surg Oncol. 2022;125(3):377–86. 10.1002/jso.26709 34617593

[13] Wang M, Li D, Chen R, Huang X, Li J, Liu Y, Liu J, Cheng W, Chen X, Zhao W (2021). Laparoscopic versus open pancreatoduodenectomy for pancreatic or periampullary tumours: a multicentre, open-label, randomised controlled trial. Lancet Gastroenterol Hepatol.

[14] Li G, Yuan L, Yu G, Xu Y, Liu J (2020). A Modified Suture Technique in Hepaticojejunostomy. Med Sci Monit.

[15] Liu Q, Zhao Z, Zhang X, Wang W, Han B, Chen X, Tan X, Xu S, Zhao G, Gao Y (2023). Perioperative and Oncological Outcomes of Robotic Versus Open Pancreaticoduodenectomy in Low-Risk Surgical Candidates: A Multicenter Propensity Score-Matched Study. Ann Surg..

[16] Jin J, Yin S-M, Weng Y, Chen M, Shi Y, Ying X, Gemenetzis G, Qin K, Zhang J, Deng X (2022). Robotic versus open pancreaticoduodenectomy with vascular resection for pancreatic ductal adenocarcinoma: surgical and oncological outcomes from pilot experience. Langenbecks Arch Surg..

[17] Wang W, Liu Q, Zhao Z-M, Tan X-L, Wang Z-Z, Zhang K-D, Liu R (2022). Comparison of robotic and open pancreaticoduodenectomy for primary nonampullary duodenal adenocarcinoma: a retrospective cohort study. Langenbecks Arch Surg.

[18] Zhang Z, Yin T, Qin T, Pan S, Wang M, Zhang H, Qin R (2022). Comparison of laparoscopic versus open pancreaticoduodenectomy in patients with resectable pancreatic ductal adenocarcinoma: A propensity score-matching analysis of long-term survival. Pancreatology..

[19] Clavien PA, Barkun J, de Oliveira ML, Vauthey JN, Dindo D, Schulick RD, de Santibañes E, Pekolj J, Slankamenac K, Bassi C (2009). The Clavien-Dindo classification of surgical complications: five-year experience. Ann Surg.

[20] de Castro SMM, Kuhlmann KFD, van Heek NT, Busch ORC, Offerhaus GJ, van Gulik TM, Obertop H, Gouma DJ (2004). Recurrent disease after microscopically radical (R0) resection of periampullary adenocarcinoma in patients without adjuvant therapy. J Gastrointest Surg..

[21] Xu S, Zhang XP, Zhao GD, Zou W-B, Zhao ZM, Hu MG, Gao YX, Tan XL, Liu Q, Liu R (2022). Robotic versus open pancreaticoduodenectomy for distal cholangiocarcinoma: a multicenter propensity score-matched study. Surg Endosc..

[22] Xu S, Zhang XP, Zhao GD, Zhao ZM, Gao YX, Hu MG, Tan XL, Liu R. Development and validation of an online calculator to predict early recurrence and long-term survival in patients with distal cholangiocarcinoma after pancreaticoduodenectomy. J Hepatobiliary Pancreat Sci. 2022;29(11):1214–25. 10.1002/jhbp.1058 34676993

[23] Vickers AJ, Holland F (2021). Decision curve analysis to evaluate the clinical benefit of prediction models. Spine J.

[24] Junrungsee S, Kittivarakul E, Ko-iam W, Lapisatepun W, Sandhu T, Chotirosniramit A (2017). Prognostic Factors and Survival of Patients with Carcinoma of the Ampulla of Vater after Pancreaticoduodenectomy. Asian Pac J Cancer Prev.

[25] Kim N, Han I, Ryu Y, Hwang D, Heo J, Choi D, Shin S (2020). Predictive Nomogram for Early Recurrence after Pancreatectomy in Resectable Pancreatic Cancer: Risk Classification Using Preoperative Clinicopathologic Factors. Cancers.

[26] Sahara K, Tsilimigras D, Toyoda J, Miyake K, Ethun C, Maithel S, Abbott D, Poultsides G, Hatzaras I, Fields R (2021). Defining the Risk of Early Recurrence Following Curative-Intent Resection for Distal Cholangiocarcinoma. Ann Surg Oncol..

[27] Yoen H, Kim JH, Hur BY, Ahn SJ, Jeon SK, Choi S-Y, Lee KB, Han JK (2021). Prediction of tumor recurrence and poor survival of ampullary adenocarcinoma using preoperative clinical and CT findings. Eur Radiol.

[28] Park HM, Park S-J, Han S-S, Hong SK, Hong EK, Kim S-W (2019). Very early recurrence following pancreaticoduodenectomy in patients with ampullary cancer. Medicine.

[29] de Castro SMM, van Heek NT, Kuhlmann KFD, Busch ORC, Offerhaus GJA, van Gulik TM, Obertop H, Gouma DJ (2004). Surgical management of neoplasms of the ampulla of Vater: local resection or pancreatoduodenectomy and prognostic factors for survival. Surgery..

[30] Sakata J, Shirai Y, Wakai T, Yokoyama N, Sakata E, Akazawa K, Hatakeyama K (2007). Number of positive lymph nodes independently affects long-term survival after resection in patients with ampullary carcinoma. European Journal of Surgical Oncology : the Journal of the European Society of Surgical Oncology and the British Association of Surgical Oncology.

[31] Schneider M, Büchler MW (2021). [Ampullary neoplasms: surgical management]. Der Chirurg; Zeitschrift Fur Alle Gebiete Der Operativen Medizen.

[32] Skórzewska M, Kurzawa P, Ciszewski T, Pelc Z, Polkowski WP (2022). Controversies in the diagnosis and treatment of periampullary tumours. Surg Oncol.

[33] Zhang X-P, Gao Y-X, Xu S, Zhao G-D, Hu M-G, Tan X-L, Zhao Z-M, Liu R (2022). A novel online calculator to predict early recurrence and long-term survival of patients with resectable pancreatic ductal adenocarcinoma after pancreaticoduodenectomy: A multicenter study. International Journal of Surgery (London, England).

