# Supplementary material for: Development and validation of web calculators to predict early recurrence and long-term survival in patients with duodenal papilla carcinoma after pancreaticoduodenectomy
Source: BMC Cancer. 2023 Nov 20;23:1129. doi: 10.1186/s12885-023-11632-5 (PMC10662559; doi:10.1186/s12885-023-11632-5)
Supplement: Supplementary file 1 — Additional file 1: Supplementary figure 1. Flowchart of our study. Supplementary table 1. Evaluated cut-off thresholds for defining DPC-ER based on the overall survival in all DPC patients (n=251). Supplementary table 2. Recurrence sites in DPC patients with ER after radical pancreaticoduodenectomy. (n=81). Supplementary table 3. Comparison of different models in predicting DPC-ER. Supplementary figure 2. The performance of the DPC-ER nomogram for predicting early recurrence (ER) compared with TNM stage in the training and validation cohorts. Supplementary table 4. The DPC-ER incidence between patients with nomogram score ≤ 63 (low-risk group) and >63 (high-risk group) in the training and the validation cohorts. Supplementary table 5. Overall survival probability and median survival time between patients with nomogram score ≤ 63 (low-risk group) and >63 (high-risk group) in training and validation cohorts. Supplementary table 6. Recurrence-free survival probability and median survival time between patients with nomogram score ≤ 63 (low-risk group) and >63 (high-risk group) in training and validation cohorts. Supplementary table 7. Comparison of different models in predicting OS of patients with DPC after PD in training and validation cohorts. Supplementary figure 4. The screenshot of DPC-ER web calculator. Supplementary figure 5. The screenshot of DPC-OS web calculator. [file 12885_2023_11632_MOESM1_ESM.docx]

# Supplementary Materials

# Supplementary figure 1. Flowchart of our study


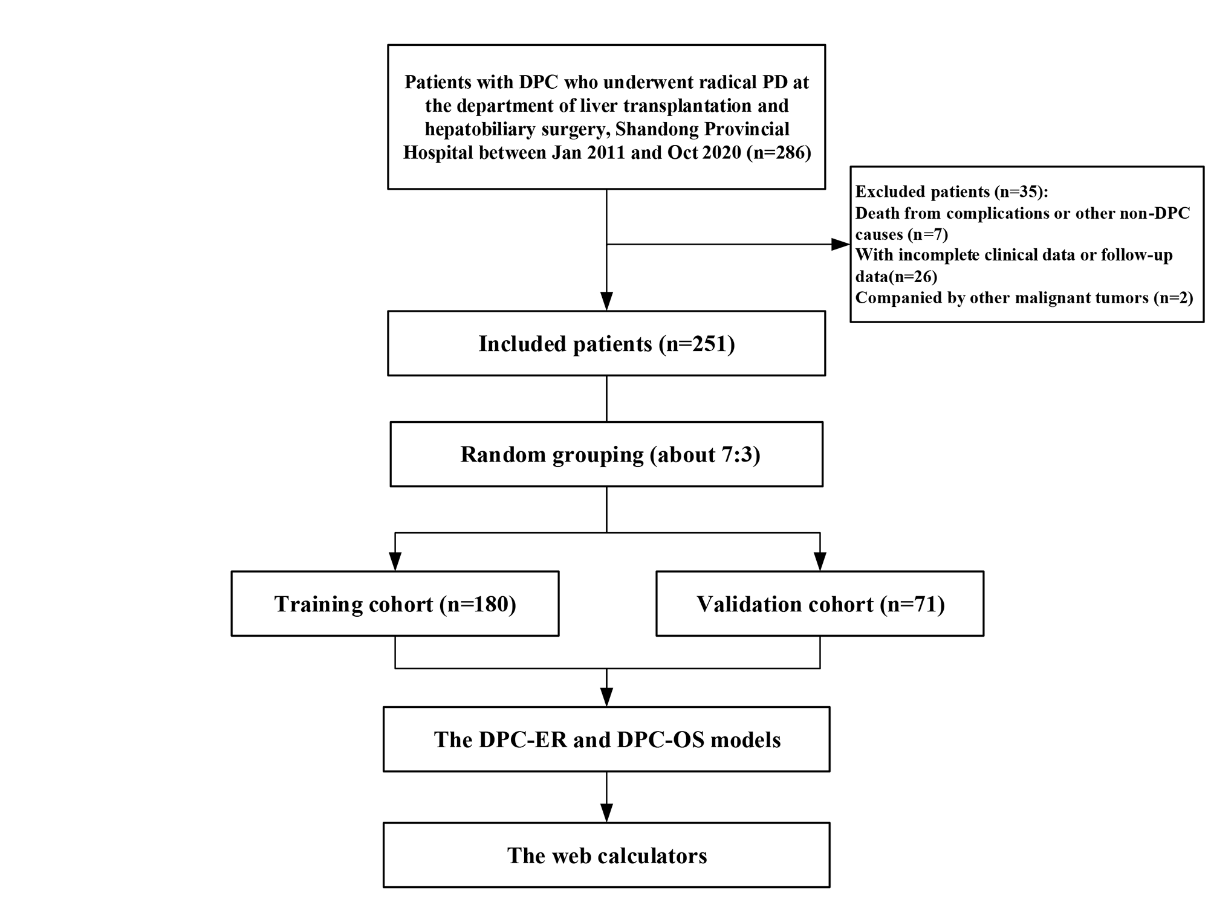


**Abbreviation:** DPC, duodenal papilla carcinoma; ER, early recurrence; OS, overall survival.

# Supplementary table 1. Evaluated cut-off thresholds for defining DPC-ER based on the overall survival in all DPC patients (n=251)

| **Evaluated cut-off** | **Potential ER group** | | **Potential non-ER group** | | ***P* value** |
| --- | --- | --- | --- | --- | --- |
|  | **N (%)** | **Median OS (95% CI), months** | **N (%)** | **Median OS (95% CI), months** |  |
| 6 months | 19 (7.6) | 12.6 (4.8-20.4) | 232 (92.4) | 52.0 (45.2-58.8) | 3.789*10^-30 |
| 12 months | 48 (19.1) | 17.5 (14.7-20.3) | 203 (80.9) | 62.0 (48.7-75.3) | 2.693*10^-41 |
| 18 months | 64 (25.5) | 20.7 (18.3-23.1) | 187 (74.5) | 65.3 (NA-NA) | 2.641*10^-38 |
| 24 months | 81 (32.3) | 21.9 (18.2-25.6) | 170 (67.7) | NA (NA-NA) | 3.596*10^-44 |
| 30 months | 99 (39.4) | 27.0 (22.4-31.5) | 152 (60.6) | NA (NA-NA) | 5.957*10^-39 |
| 60 months | 122 (48.6) | 32.1 (28.1-36.1) | 129 (51.4) | NA (NA-NA) | 7.482*10^-25 |

Abbreviation: DPC, duodenal papilla carcinoma; ER, early recurrence; OS, overall survival; CI, confidence interval; NA, not available.

# Supplementary table 2. Recurrence sites in DPC patients with ER after radical pancreaticoduodenectomy. (n=81)

| Recurrence sites † | N (%) |
| --- | --- |
| Local, N (%) | 16 (19.8) |
| Liver, N (%) | 39 (48.1) |
| Distant lymph node, N (%) | 9 (11.1) |
| Peritoneum, N (%) | 7 (8.6) |
| Lung, N (%) | 2 (2.5) |
| Bone, N (%) | 3 (3.7) |
| Uncertain, N (%) | 9 (11.1) |

†, duplications present; DPC, duodenal papilla carcinoma; ER, early recurrence.

# Supplementary table 3. Comparison of different models in predicting DPC-ER

| **Models in cohorts** | **AUC (95% CI)** | ***P* value** |
| --- | --- | --- |
| Training cohort |  |  |
| The DPC-ER nomogram | 0.759 (0.685-0.832) | Reference |
| Tumor size | 0.613 (0.524-0.703) | 0.002 |
| Perineural invasion | 0.585 (0.491-0.679) | <0.001 |
| N stage | 0.620 (0.527-0.712) | <0.001 |
| Differentiation | 0.653 (0.568-0.738) | 0.004 |
| Validation cohort |  |  |
| The DPC-ER nomogram | 0.729 (0.601-0.856) | Reference |
| Tumor size | 0.619 (0.476-0.762) | 0.049 |
| Perineural invasion | 0.615 (0.472-0.757) | 0.041 |
| N stage | 0.582 (0.440-0.725) | 0.025 |
| Differentiation | 0.577 (0.437-0.716) | 0.041 |

**Abbreviation:** DPC, duodenal papilla carcinoma; ER, early recurrence; AUC, area under receiver operating characteristic (ROC) curve; CI, confidence interval.

**Supplementary figure 2. The performance of the DPC-ER nomogram for predicting early recurrence (ER) compared with TNM stage in the training and validation cohorts.**


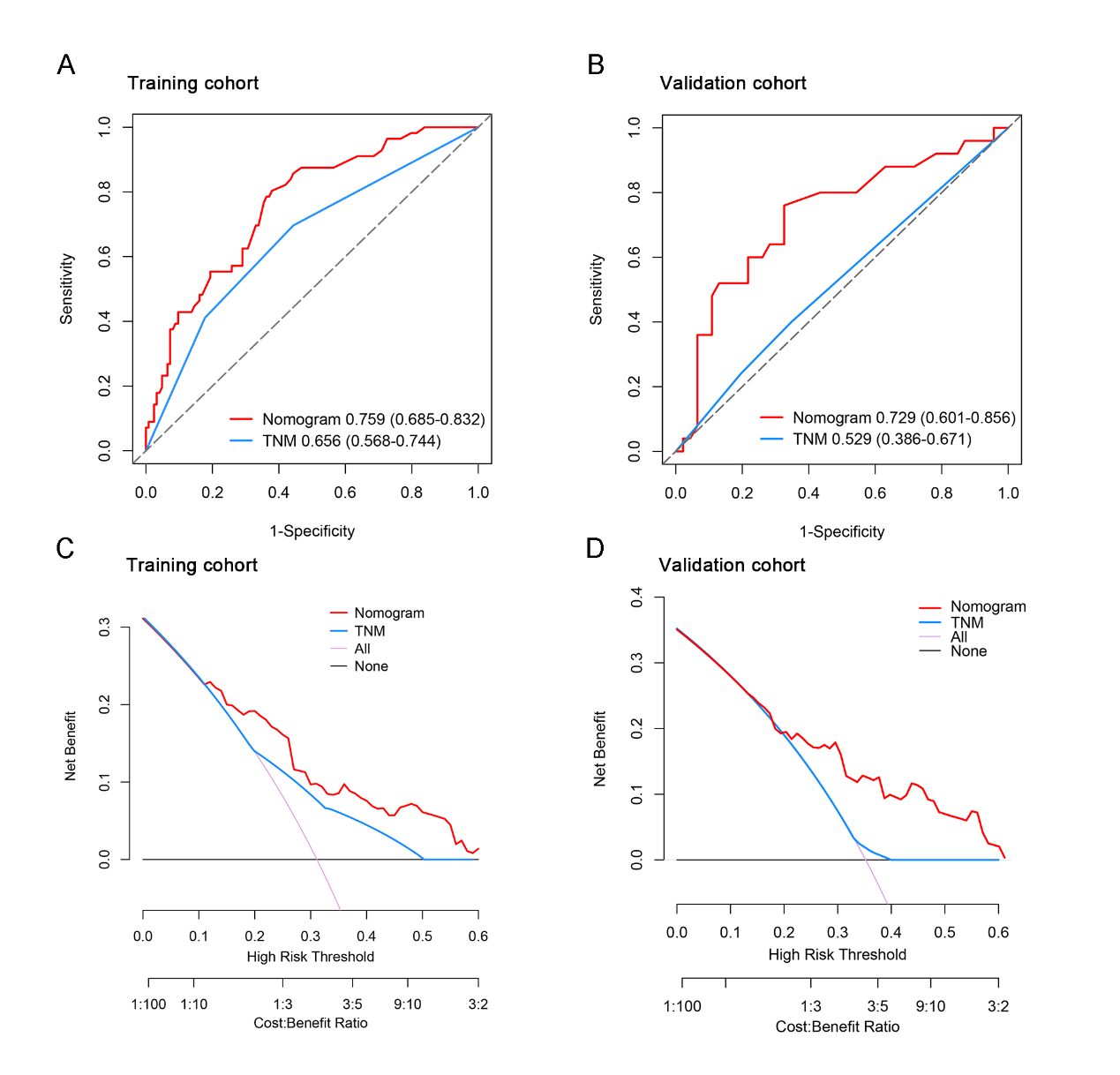


A-B, receiver operating characteristic (ROC) curve analyses in the training and the validation cohorts, respectively; C-D, decision curve analysis (DCA) in the training and the validation cohorts, respectively.

# Supplementary table 4. The DPC-ER incidence between patients with nomogram score ≤ 63 (low-risk group) and >63 (high-risk group) in the training and the validation cohorts

| **Cohorts** | **Low-risk group** | **How-risk group** | ***P* value** |
| --- | --- | --- | --- |
| Training cohort |  |  |  |
| Non-ER | 77 (87.5) | 47 (51.1) | <0.001 |
| ER | 11 (12.5) | 45 (48.9) |  |
| Validation cohort |  |  |  |
| Non-ER | 27 (77.1) | 19 (52.8) | 0.032 |
| ER | 8 (22.9) | 17 (47.2) |  |

**Abbreviation:** DPC, duodenal papilla carcinoma; ER, early recurrence;

# Supplementary table 5. Overall survival probability and median survival time between patients with nomogram score ≤ 63 (low-risk group) and >63 (high-risk group) in training and validation cohorts

| **Cohorts** | **Time after PD** | **Low-risk group** | **High-risk group** | ***P* value** |
| --- | --- | --- | --- | --- |
| Training cohort (n=180) | 1-year | 100.0% | 90.0% | <0.001 |
|  | 3-year | 87.6% | 50.7% |  |
|  | 5-year | 66.0% | 21.6% |  |
|  | Median OS (month) | NA (NA-NA) | 39.0 (29.7-48.3) |  |
| Validation cohort (n=71) | 1-year | 100.0% | 93.9% | 0.030 |
|  | 3-year | 68.6% | 46.4% |  |
|  | 5-year | 40.0% | 30.9% |  |
|  | Median OS (month) | 53.2 (35.4-71.0) | 30.2 (6.8-53.6) |  |

**Abbreviation:** PD, pancreaticoduodenectomy; OS, overall survival.

# Supplementary table 6. Recurrence-free survival probability and median survival time between patients with nomogram score ≤ 63 (low-risk group) and >63 (high-risk group) in training and validation cohorts

| **Cohorts** | **Time after PD** | **Low-risk group** | **High-risk group** | ***P* value** |
| --- | --- | --- | --- | --- |
| Training cohort (n=180) | 1-year | 92.8% | 69.9% | <0.001 |
|  | 3-year | 72.8% | 30.1% |  |
|  | 5-year | 51.1% | 19.6% |  |
|  | Median RFS (month) | 62.5 (NA-NA) | 23.4 (18.8-28.0) |  |
| Validation cohort (n=71) | 1-year | 96.9% | 65.5% | 0.021 |
|  | 3-year | 55.7% | 26.5% |  |
|  | 5-year | 0.0% | 26.5% |  |
|  | Median RFS (month) | 37.5 (31.3-43.7) | 14.5 (1.3-27.7) |  |

**Abbreviation:** PD, pancreaticoduodenectomy, RFS, recurrence-free survival; NA, not available.

#
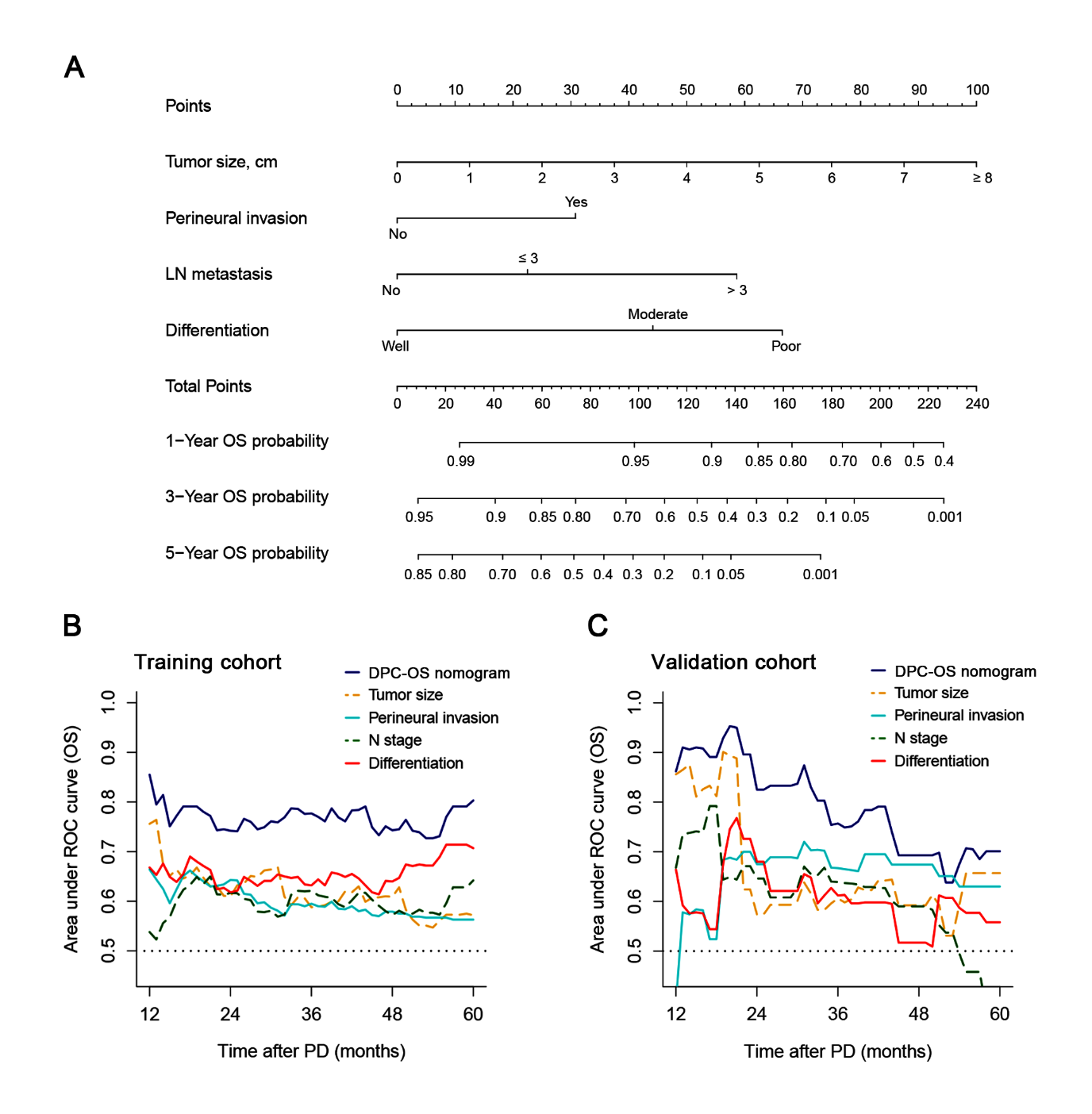
Supplementary figure 3. The DPC-OS nomogram

The DPC-OS nomogram (A) and timeROCs of DPC-OS nomogram and single variable in the training and validation cohorts (B-C). DPC, duodenal papilla carcinoma; OS, overall survival; ROC, receiver operating characteristic curve.

# Supplementary table 7. Comparison of different models in predicting OS of patients with DPC after PD in training and validation cohorts

| **Models in cohorts** | **Time-dependent AUCs for OS** | | | | | | | ***P* value** |
| --- | --- | --- | --- | --- | --- | --- | --- | --- |
|  | **Minimum** | **1-Year** | **2-Year** | **3-Year** | **4-Year** | **5-Year** | **Maximum** |  |
| Training cohort |  |  |  |  |  |  |  |  |
| The DPC-OS nomogram | 0.666 | 0.848 | 0.666 | 0.741 | 0.740 | 0.795 | 0.848 | Ref |
| Tumor size | 0.542 | 0.818 | 0.564 | 0.595 | 0.601 | 0.572 | 0.818 | <0.001 |
| Perineural invasion | 0.560 | 0.658 | 0.617 | 0.596 | 0.583 | 0.567 | 0.658 | <0.001 |
| N stage | 0.510 | 0.527 | 0.580 | 0.604 | 0.599 | 0.649 | 0.649 | <0.001 |
| Differentiation | 0.583 | 0.669 | 0.583 | 0.597 | 0.633 | 0.704 | 0.711 | <0.001 |
| Validation cohort |  |  |  |  |  |  |  |  |
| The DPC-OS nomogram | 0.638 | 0.862 | 0.825 | 0.757 | 0.693 | 0.701 | 0.953 | Ref |
| Tumor size | 0.531 | 0.856 | 0.574 | 0.606 | 0.592 | 0.657 | 0.901 | <0.001 |
| Perineural invasion | 0.397 | 0.397 | 0.675 | 0.667 | 0.674 | 0.630 | 0.720 | <0.001 |
| N stage | 0.382 | 0.668 | 0.646 | 0.639 | 0.590 | 0.382 | 0.792 | <0.001 |
| Differentiation | 0.509 | 0.664 | 0.680 | 0.612 | 0.517 | 0.558 | 0.768 | <0.001 |

**Abbreviation:** OS, overall survival; DPC, duodenal papilla carcinoma; PD, pancreaticoduodenectomy; AUC, area under the time-dependent receiver operating characteristic (ROC) curve.

**Supplementary figure 4. The screenshot of DPC-ER web calculator**


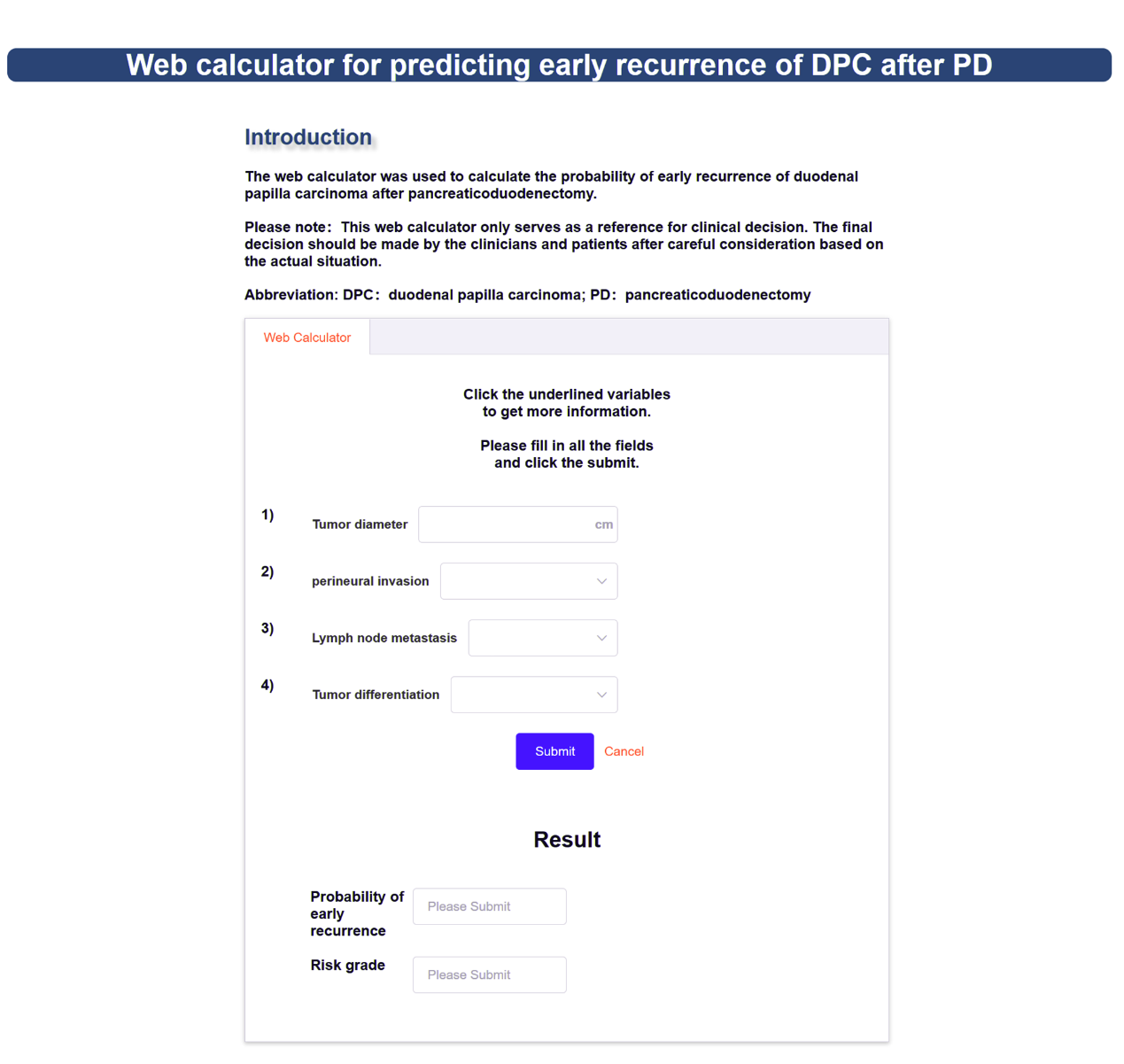


DPC, duodenal papilla carcinoma; ER, early recurrence.

**Supplementary figure 5. The screenshot of DPC-OS web calculator**


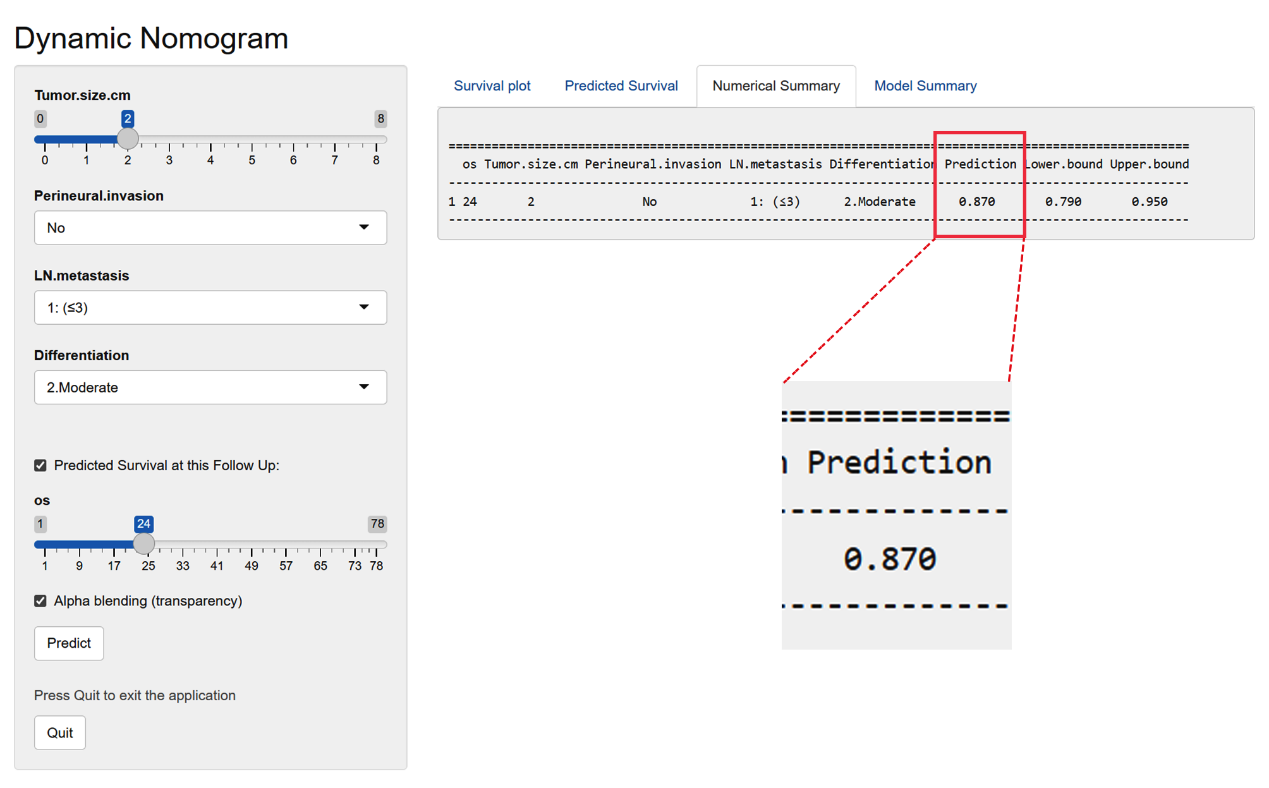


DPC, duodenal papilla carcinoma; OS, overall survival.
